# Sustainability of the Aging Network's COVID-19 Responses: Isolated Impact or Transformative Change?

**DOI:** 10.1093/geroni/igab046.1792

**Published:** 2021-12-17

**Authors:** Suzanne Kunkel, Suzanne Kunkel

## Abstract

The COVID-19 pandemic mobilized individuals, organizations, and communities. Area Agencies and Aging (AAAs) and Title VI Native American Programs, core organizations of the network of community-based organizations (CBOs) that serve older adults and their families, pivoted their service delivery methods to provide life-sustaining services. Their long-standing expertise in community needs assessment, pre-existing cross-sectoral partnerships, and an infusion of emergency federal funding, enabled this rapid response. Recently concluded studies using key-informant interviews and national surveys of AAAs and Title VI programs highlight these service adaptations, from expansion of home-delivered meal programs to new partnerships in telehealth. These organizations also reported expansion of services offered and people served, and the emergence or strengthening of partnerships with other CBOs, businesses, and governmental organizations such as public health entities. For example, 78% of the respondents to the recent survey of AAAs reported that they have a role in vaccination outreach, scheduling support, or delivery. The papers in this symposium will use these new studies to describe the nature, origins, and potential sustainability of new and expanded services and partnerships. The Collective Impact Model for community change (introduced in the Stanford Social Innovation Review) will provide a framework for the discussion. Built on the importance of cross-sector coordination, the five pillars of success for collective rather than isolated impact are: a common agenda, mutually reinforcing activities, continuous communication, shared measurement, and a backbone organization. Each of these five pillars is relevant to the heightened community response during the pandemic, and to the likelihood of sustainability.

